# Crop changes from the XVI century to the present in a hill/mountain area of eastern Liguria (Italy)

**DOI:** 10.1186/1746-4269-5-9

**Published:** 2009-04-11

**Authors:** Rodolfo Gentili, Elio Gentili, Sergio Sgorbati

**Affiliations:** 1 Dipartimento di Scienze dell'Ambiente e del Territorio, Università degli Studi di Milano-Bicocca, Piazza della Scienza n. 1, I-20126, Milano, Italy; 2 Biblioteca Niccolò V, Archivi Vescovili Lunensi, Via Mascardi n. 93, I-19038 – Sarzana (SP), Italy

## Abstract

**Background:**

Chronological information on the composition and structure of agrocenoses and detailed features of land cover referring to specific areas are uncommon in ethnobotanical studies, especially for periods before the XIX century. The aim of this study was to analyse the type of crop or the characteristics of soil cover from the XVI century to the present.

**Methods:**

This diachronic analysis was accomplished through archival research on the inventories of the Parish of St. Mary and those of the Municipality of Pignone and from recent surveys conducted in an area of eastern Liguria (Italy).

**Results:**

Archival data revealed that in study area the primary means of subsistence during the last five centuries, until the first half of the XX century, was chestnuts. In the XVIII and XIX centuries, crop diversification strongly increased in comparison with previous and subsequent periods. In more recent times, the abandonment of agricultural practices has favoured the re-colonisation of mixed woodland or cluster-pine woodland.

**Conclusion:**

Ancient documents in the ecclesiastic or municipal inventories can be a very useful tool for enhancing the knowledge of agricultural practice, as well as of subsistence methods favoured by local populations during a particular time and for reconstructing land use change over time.

## Background

One frequent problem in assessing the causes and degree of changes in land use and crop diversity through time has been the scarcity of historical data for documenting such changes [1]. Unfortunately, archival documentation has either not been preserved or is difficult to find, especially pertaining to periods prior to the XIX century.

The Council of Trent (1545–1563), introduced the bishops' requirement for periodic inspection of the inventories in each parish, convent or other ecclesiastic institution (including cultivated fields, forests, mills, etc.), encouraging their compilation [2]. Such documents, used rarely in ethnobotanical investigation up to now, can be a very important source of chronological information on the composition and/or structure of agrocenoses and detailed features of land cover when specific for certain areas or even toponyms [3]. Therefore, examination of the ecclesiastic possession inventories can enhance the knowledge of agricultural and livestock practice and subsistence methods of local populations during this time. In addition, other historical sources, such as cadastral maps, tax registers and manorial accounts, can give useful information about agricultural practices [4]. Furthermore, indirect data regarding the natural environment (i.e. land cover) can be extrapolated from ancient archival documents.

It is well known that in past centuries subsistence agriculture was the main source of food for European people. Everywhere, cereals were the most important crop. Locally, cereals were partially replaced by other farm products, such as potatoes, legumes, olives, and chestnuts [5].

During the second half of the XX century, in many hill/mountain regions of Europe (especially those close to the Mediterranean Sea) abandonment of mall-scale or household agriculture increased [6]. This abandonment occurred as humans began to systematically use extensive farming techniques, mostly in planned areas, and under the impacts of industrial/economic development [7].

Time-series investigations of changes in crop variety, even on a small scale, might uncover insights with broader regional implications [8].

In this article, we attempt to reconstruct changes in the type of crops and the characteristics of soil cover during the time from the XVI century to the present through archival and field research conducted on the inventories of the Parish of St. Mary and the Pignone territory in eastern Liguria.

## Methods

### Study area

**Figure 1:**
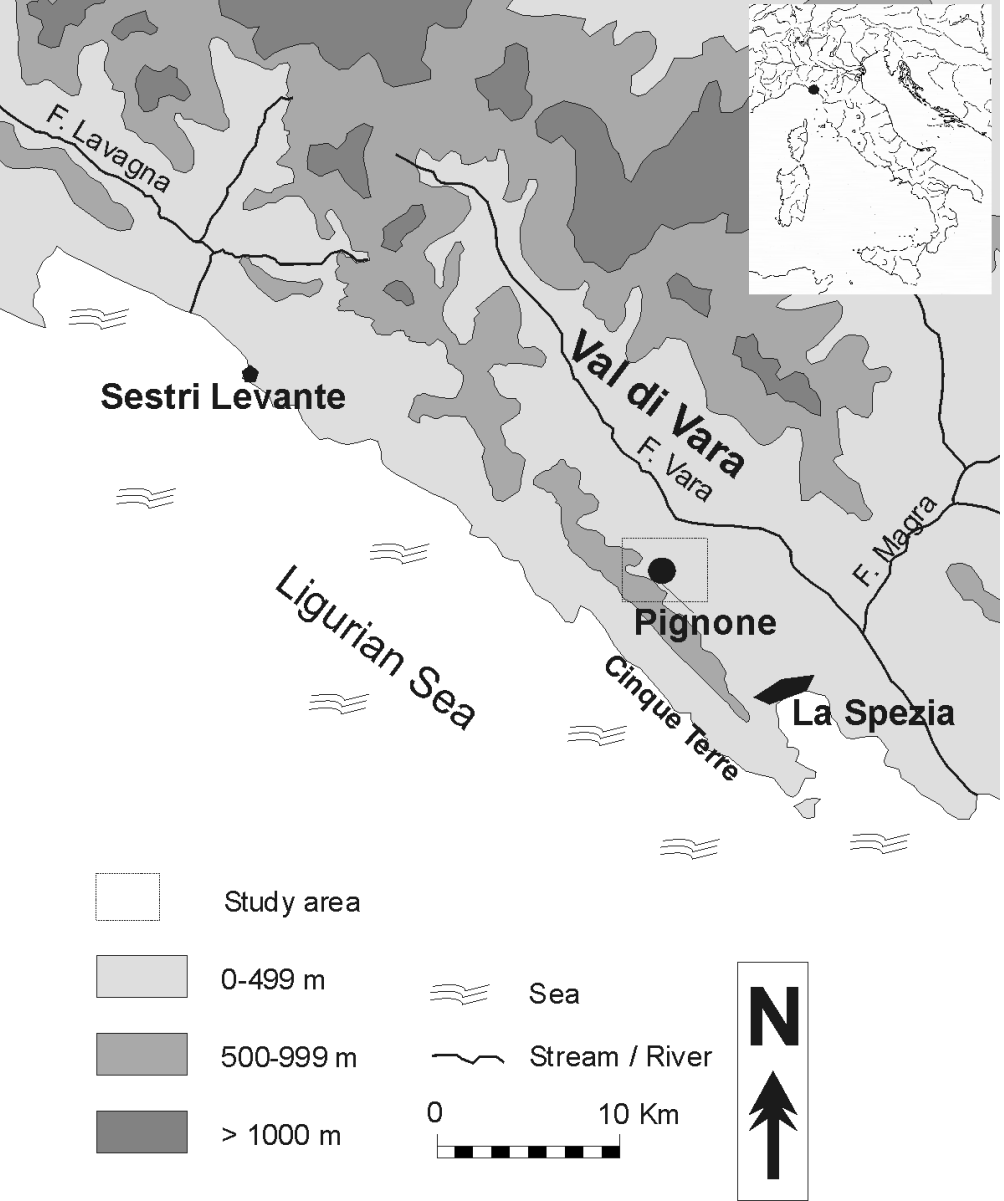
**Study area**. The study area.

The Municipality of Pignone lies in eastern Liguria, in Val di Vara, at the border with the National Park of the Cinque Terre, less than 10 kilometres from the Ligurian Sea (Figure 1); it rises 189 m above sea level. The highest peak in the area reaches about 600 meters. In the study area, a Site of Community Importance (SCI), related to a karstic zone, is present.

From a historical point of view, the origin of Pignone village is very ancient, as indicated by archaeological findings from the Bronze Age on Mount Castellaro [9]. The same name, "Castellaro", indicates the presence of Ligurian population settlements. In the Middle Ages, this area was the domain of the Malaspina dynasty, the bishops of Luni, the Fieschi dynasty and the Genova Republic [10].

The climate of the study area is sub-Mediterranean and characterised by warm summers and temperate winters; precipitation is mostly concentrated in spring and autumn.

From a phytogeographic point of view, the study area is located in the Italo-Thyrrenian province, within the Mediterranean region [11].

The natural vegetation present in the Pignone territory mainly consists of holm-oak (*Quercus ilex *L.) mixed woodland with a high cover of downy-oak (*Quercus pubescens *Willd.). In a wide portion of the territory, cluster-pine (*Pinus pinaster *Aiton) anthropogenic and semi-natural (favoured by fire) woodlands are present. On fresh slopes, a mixed woodland dominated by European hop-hornbeam (*Ostrya carpinifolia *Scop.), manna-ash (*Fraxinus ornus *L.) and downy-oak is present. Locally, the vegetation is characterised by anthropogenic woods of chestnut (*Castanea sativa *Mill.). Black-alder (*Alnus glutinosa *(L.) Gaertn.) woodlands grow at the bottom of valleys, along with willow (*Salix *spp.) formations.

### Archival data and field survey

In this study, five time periods since the XVI century are compared according to their crop or land use in the Pignone territory in the following years: 1588, 1663, 1763, 1828, 1878, 1933, and 2008 (Table 1, Table 2). The types of crops have been obtained through documentary investigation of all archival folders existing relating to the Parish of St. Mary in Pignone and to the Municipality of Pignone. The former are stored in the "Niccolo V" Lunense Episcopal Archive of Sarzana (SP): series "Filze Parrocchiali" [12-15]. The latter are stored in the State Archive of La Spezia [16,17].

**Table 1:** Inventories (part A)

**Type of crop**							
**Toponym**	**1588**	**1663**	**1763**	**1828**	**1878**	**1933**	**2008**

*A piè di Cravadora*	Cg	Cg	Cg	Cg, W	x	x	Cg, W
*Alla Focina*	M	x	x	x	x	x	W
*Alla Presa*	x	Hf	x	x	x	Cg	x
*Aretta*	M	Hf	M, Ar	Al, Vy, Ar	x	x	W
*Borela*	x	x	x	Cg	x	W	B, W
*Calcinarla 1*	M	Hf	x	Al, Vy, Ar	Al, Vy, Ar	x	B
*Calcinarla 2*	M, Hf	Hf	M, Ar	Al, Vy, Ar	Al, Vy	Al, Vy	B, Al
Campedio*	M	Og	x	x	x	x	x
*Campuscasco 1*	x	Cg	x	x	Cg, W	Cg, W	W
Canavaro*	x	Hf	Hf	Al, Vy, Ar	x	Vy, Al	x
*Carpena*	Cg	x	x	x	x	x	x
Coloreda 1*	Cg	Cg	Cg	Cg, W	x	W	Cg, W
*Corasca*	x	Cg	x	x	x	x	x
*Coroni*	Cg	x	x	x	x	x	x
*Cravadora grande 1*	Cg	Cg	Cg	Cg, W	Cg	W	W
*Cravadora grande 2*	Cg	Og	x	Cg, W	Cg, W	Cg, W	W
*Cravadora minore 1*	Cg	Cg	Cg	x	Cg	Cg, W	W
*Cusanasco*	x	Og	x	Al, Vy, Ar	x	x	x
*Favà 1*	Cg	Cg	Cg	Cg	Cg	Cg	Cg, W
Fosicchia 1*	Cg, F	x	x	Cg, W	Cg	x	x
*Il Castellaro 1*	M	Og	Og	Og	Og, Cg	x	W
*Il Castellaro di Bado*	x	x	x	Og, Cg	x	x	W
*Il Piaggio 1*	x	Og	M, Ar	Cg, Al, W	M	M	W
*Il Piaggio 2*	x	Og	Og, Cg	Og, Cg	Og, Al	Al	W
*Il Piaggio 3*	x	x	x	Og, Cg, W	Cg	Al, Vy	W
*Illegible toponym*	M	x	x	x	x	x	x
*Illegible toponym*	x	Cg	x	x	x	x	x
*Illegible toponym*	x	x	Al, Ar	x	x	x	x
*Illegible toponym*	x	x	Cg	x	x	x	x
*La Chiappara*	Cg	x	Cg	x	Cg	x	x
*La Chiosa 1*	M, Vy, Ar	Vy	Cg, Ar, Vy, Al	Cg, Ar, Vy, Al	x	x	B, Al
*La Chiosa 2*	x	x	x	Cg	x	x	W
*La dal Canale*	x	x	x	Cg, W	Cg	Al, Vy	x
*La Fornace*	x	x	x	Al, Vy, Ar	x	x	x
*La Giara*	Hf*	Vy*	x	Al, Vy, Ar	x	Al	x
*La Ligia*	x	x	x	Cg, W	x	W, Cg	W
*La Prata*	x	x	x	Al, Vy, Ar	Al	x	x
*La Ripalta*	Cg	x	x	Cg, W	x	x	x
*La Riva*	x	x	x	Al, Vy, Ar	Al, Vy	x	x
*Linà*	x	Hf	Al, Ar	Cg	x	W	x
*Linà*	x	Og	x	x	x	x	x
Madonna del Ponte 1*	x	Cg	Cg	Cg, W	x	W	W
*Maggioli*	x	x	x	Al, Vy, Ar	x	x	x
*Nel Piano*	M	x	x	x	Cg, M	x	x
*Nel Trezzo*	M	x	x	x	x	x	x
*Patina*	x	x	x	Cg	x	x	x
*Pezza del Brazo*	x	x	x	Al, Vy, Ar	x	x	x
*Pezza del Molino*	x	x	x	Al, Vy, Ar	x	Al	W
Pezzo Grande 1*	M, Vy, Ar	x	x	Al, Vy, Ar	Vy, M, Ar, Al	x	x
*Pian de Fossa 1*	Cg	Cg	Cg	Cg, W	Cg	Cg	W
*Piè di Favà*	M	x	M, Ar	x	x	x	W
*Piè di Favà*	M*	x	M, Ar	x	x	x	W
*Pignon soprano 1*	M, Vy, Ar	Hf	Cg, Ar, Vy, Al	Cg, Ar, Vy, Al	x	Al, Vy, Cg	x
*Pignon soprano 2*	x	x	Cg, Ar, Vy, Al	x	x	Al, Vy, Cg	x
*Pignon soprano 3*	x	x	Cg, Ar, Vy, Al	x	x	x	x
*Pignon soprano 4*	x	x	Cg, Ar, Vy, Al	x	x	x	x
*Pignon soprano 5*	x	x	Cg, Ar, Vy, Al	x	Al	x	x
*Pignon soprano 6*	x	x	M	x	M	x	x
*Pignon soprano 7*	x	x	Og, Cg	x	x	x	x
*Pilora 1*	x	x	x	Al, Vy, Ar	x	Og	Og
*Postemi*	x	Cg	x	x	x	x	x
*Rezzo 1*	Cg	x	Ar	x	x	x	x
*Rezzo 2*	G	x	x	x	x	x	x
*Rezzo 3*	G*	Vy*	x	x	x	x	x
*Rezzo 4*	M	x	Ar	x	x	x	x
*Rezzo 5*	M	x	Ar	x	x	x	x
*S. Antonio 1*	x	x	x	Al, Vy, Ar	x	Og	W, Og
*S. Michele*	x	x	M, Ar	Al, Vy, Ar	x	x	x
Scandolara 1*	Cg	Cg	Cg	x	x	Cg	W
*Sotto la Lama*	x	x	x	Al, Vy, Ar	x	x	x
*Spinzo 1*	x	Og	x	x	x	x	x
*Spinzo 2*	x	Og	x	x	x	x	x
*Spinzo 3*	x	Og	x	x	x	x	x
*Sprugola A*	Cg	x	Cg, M	x	x	x	x
*Sprugola B*	Cg	x	Cg	x	x	x	x
*Su dal Canale*	x	x	x	Cg, W	x	x	x
*Tra la Chiesa*	M	x	Og, Cg	x	x	x	W, Vy
*Tra le case*	M	Hf	M	x	x	x	x
*Va' 1*	x	x	x	Cg	Cg	Cg	x
*Vaccheredo*	Cg	x	x	x	x	x	x

Inventories, ordered for every toponym, of the Parish of St. Mary and of the Municipality of Pignone, for the years 1588, 1663, 1763, 1828, 1878, 1933 and recent times. Part A comprises toponyms listed in the ecclesiastic inventories (years 1588, 1663, 1763, 1828). Legend: Ar = arboretum; B = buildings; Cg = chestnut grove; Hf = hempfield; G = grassland; M = meadow; Og = olive grove; Al = arable land; W = woodland; Vy = vineyard; * = include a mill; x: no data.

**Table 2:** Inventories (part B)

**Type of crop**							
**Toponym**	**1588**	**1663**	**1763**	**1828**	**1878**	**1933**	**2008**

*Ai Laghi 1*	x	x	x	x	W	Al	Al
*Ai Laghi 2*	x	x	x	x	x	Al, Vy	Al, Vy
*Alle Noci*	x	x	x	x	x	W	W
*Bandita*	x	x	x	x	x	W	W
*Banzola 1*	x	x	x	x	Cg	W, Cg	W
*Banzola 2*	x	x	x	x	W	W	W
*Banzola 3*	x	x	x	x	W	W	W
*Banzola 4*	x	x	x	x	x	W	W
*Battipagliano 1*	x	x	x	x	Cg	Cg	Cg
*Battipagliano 2*	x	x	x	x	Al	Al	x
*Battipagliano 3*	x	x	x	x	Al, Cg	Cg, M	Cg, W
*Battipagliano 4*	x	x	x	x	Vy, Al	Al	W
*Battipagliano 5*	x	x	x	x	Vy, Al	Al	W
*Battipagliano 6*	x	x	x	x	x	Al	Al
*Battipagliano 7*	x	x	x	x	x	Al, M	Al, M
*Bogiolo*	x	x	x	x	x	Al	Al
*Borale 1*	x	x	x	x	Vy, Al	Al	Al
*Borale 2*	x	x	x	x	Vy, Al, Og	x	W
*Borale 3*	x	x	x	x	Vy, Al	x	W
*Borale 4*	x	x	x	x	Al, Vy	x	M
*Borale 5*	x	x	x	x	Cg	x	B, W
*Bosco del Gallino*	x	x	x	x	W	x	W
*Calcinarla 3*	x	x	x	x	W	x	B, Al
*Calcinarla 4*	x	x	x	x	Al, Vy, irrig	Al	B, Al
*Calcinarla 5*	x	x	x	x	Cg	x	B, Al
*Calcinarla 6*	x	x	x	x	Al, Vy	Al, Vy, Cg	B, Al
*Calcinarla 7*	x	x	x	x	Al, Vy	Al	B, Al
*Calcinarla 8*	x	x	x	x	Al, Vy	x	B, Al
*Calcinarla 9*	x	x	x	x	Al, Vy	x	B, Al
*Campuscasco 2*	x	x	x	x	Cg	x	W
*Cantarana*	x	x	x	x	Al, Vy	x	x
*Castellino*	x	x	x	x	x	W	x
*Cerreta*	x	x	x	x	x	Cg, W	x
*Cerreta*	x	x	x	x	Og	W	x
*Ciazzo*	x	x	x	x	Vy, Al	x	x
*Colorede 2*	x	x	x	x	W	W	Cg, W
*Costa dell'Olivella*	x	x	x	x	Cg	x	W
*Cravadora 1*	x	x	x	x	Cg	Cg, W	W
*Cravadora 2*	x	x	x	x	W	W	W
*Cravadora 3*	x	x	x	x	Cg	Cg	W
*Cravadora 4*	x	x	x	x	Cg	W	W
*Cravadora 5*	x	x	x	x	Cg	W	W
*Cravadora 6*	x	x	x	x	Cg	W	W
*Cravadora 7*	x	x	x	x	Cg	Cg, W	W
*Cravadora di Sotto*	x	x	x	x	W, Cg	W	W, Cg
*Cravadora minore 2*	x	x	x	x	Cg	W	W
*Cuccaro 1*	x	x	x	x	Al, Vy, Ar	W	W
*Cuccaro 10*	x	x	x	x	Cg	Cg	Cg
*Cuccaro 2*	x	x	x	x	Al	W	W
*Cuccaro 3*	x	x	x	x	Cg	W	W, Cg
*Cuccaro 4*	x	x	x	x	Al, Vy, Ar	Al, Vy, Cg	Al
*Cuccaro 5*	x	x	x	x	Al	Al, Vy	Vy
*Cuccaro 6*	x	x	x	x	Al	W	W
*Cuccaro 7*	x	x	x	x	Cg	Cg, W	W
*Cuccaro 8*	x	x	x	x	Al, Vy	Al, Vy	Al, Vy
*Cuccaro 9*	x	x	x	x	Al	W	W
*Cuneo 1*	x	x	x	x	x	Al, W	Al, W
*Cuneo 2*	x	x	x	x	x	W	x
*Cuneo sottano 1*	x	x	x	x	x	Al	x
*Cuneo sottano 2*	x	x	x	x	x	W, Al	x
*Cusidore*	x	x	x	x	W	x	W
*Due Canali 1*	x	x	x	x	x	Cg	x
*Due Canali 2*	x	x	x	x	x	Cg	x
*Due Canali 3*	x	x	x	x	x	Cg	x
*Favà 2*	x	x	x	x	Cg, Og	Cg	Cg, W
*Favà 3*	x	x	x	x	Cg, Og	W	W
*Filagne*	x	x	x	x	Al	x	x
*Fontanella 1*	x	x	x	x	Cg, W	x	Cg, W
*Fontanella 2*	x	x	x	x	Cg, W	x	Cg, W
*Fontanella 3*	x	x	x	x	W	x	W
Fosicchia 2*	x	x	x	x	Cg	Cg	x
Fosicchia 3*	x	x	x	x	Cg	Cg	x
*Fossalonga*	x	x	x	x	Cg	x	x
*Foxina 1*	x	x	x	x	M	Al	x
*Foxina 2*	x	x	x	x	Al, Vy	Al	x
*Foxina 3*	x	x	x	x	x	Al	x
*Foxina 4*	x	x	x	x	x	Al	x
*Foxina Grande*	x	x	x	x	x	Al, Vy	x
*Frantoio 1*	x	x	x	x	x	Al, Vy	x
*Frantoio 2*	x	x	x	x	x	Al, Vy	x
*Frantoio 3*	x	x	x	x	x	Al	x
*Frantoio 4*	x	x	x	x	x	Al	x
*Frassaneda*	x	x	x	x	x	W	x
*Gaggiolo*	x	x	x	x	Cg, Al	W	x
*Garzarelli*	x	x	x	x	x	W	x
*Grizzola 1*	x	x	x	x	Vy, Ar, Al	W, Cg	W, Cg
*Grizzola 2*	x	x	x	x	Cg, Og	Al, Vy, Og, Cg	W, Cg
*Grizzola 3*	x	x	x	x	Al	Cg	W, Cg
*Grizzola 4*	x	x	x	x	Cg	Cg	W, Cg
*Grizzola 5*	x	x	x	x	Cg	Cg	W, Cg
*Grizzola 6*	x	x	x	x	Cg, Og, Al	Cg	W
*Grizzola 7*	x	x	x	x	x	Al, Vy, Og	Al, Vy, Og
*Il Campo 1*	x	x	x	x	Vy, Ar, Al	Al	x
*Il Campo 2*	x	x	x	x	Vy, Ar, Al	Al, Vy	x
*Il Campo 3*	x	x	x	x	Al	Al	x
*Il Campo 4*	x	x	x	x	Vy, Al, Cg	Cg	x
*Il Castellaro 2*	x	x	x	x	Og	x	W
*Il Giardino*	x	x	x	x	Al, Vy	x	x
*Il Piaggio 4*	x	x	x	x	Al	Al	x
*Il Piaggio 5*	x	x	x	x	x	Al	x
*Il Piaggio 6*	x	x	x	x	x	Og, Al	x
*Lama di Pianello*	x	x	x	x	Cg, W	x	x
Madonna del Ponte 2*	x	x	x	x	Cg	Cg	W
Madonna del Ponte 3*	x	x	x	x	Cg	x	W, Cg
*Migiaro*	x	x	x	x	Cg	x	W, Cg
*Migliarese 1*	x	x	x	x	Cg	Cg	W, Cg
*Migliarese 2*	x	x	x	x	Cg	Cg	W, Cg
*Migliarese 3*	x	x	x	x	x	W	W
*Migliarese 4*	x	x	x	x	x	Cg	W
*Migliarese 5*	x	x	x	x	x	Cg	W
*Migliarese 6*	x	x	x	x	x	W	W
*Migliarese 7*	x	x	x	x	x	W	W
*Migliarese 8*	x	x	x	x	x	W	W
*Migliarese di sopra*	x	x	x	x	x	W	W
*Migliarese di sotto*	x	x	x	x	x	Cg, W	W
*Monteletto 1*	x	x	x	x	Og, Vy, Al	M	x
*Monteletto 2*	x	x	x	x	Og, Vy, Al	x	x
*Monteletto 3*	x	x	x	x	Vy, Og, Cg	x	x
*Monteletto 4*	x	x	x	x	Cg	x	x
*Mostani*	x	x	x	x	Cg, W	x	x
*Narneia Grande 1*	x	x	x	x	x	W	x
*Narneia Grande 2*	x	x	x	x	x	W	x
*Olivella*	x	x	x	x	Cg	x	W
*Orto 1*	x	x	x	x	x	Al	x
*Orto 2*	x	x	x	x	x	Al, Vy	x
*Orto 3*	x	x	x	x	x	Al, Vy	x
*Pastine 1*	x	x	x	x	Al, Vy	Al, Vy	Al, Vy
*Pastine 2*	x	x	x	x	Cg	Vy, Cg	Cg
*Pastine 3*	x	x	x	x	Cg	Vy, Al	Vy, Al
*Pastine 4*	x	x	x	x	Cg, W	x	Cg, W
*Pastine 5*	x	x	x	x	Vy, Ar, Al	x	Cg, W
*Pastine 6*	x	x	x	x	x	Al, Vy, Cg	Cg, W
*Perlo 1*	x	x	x	x	Og, Cg	Og, Cg	W
*Perlo 2*	x	x	x	x	x	Al	W, Cg
*Perlo 3*	x	x	x	x	x	W, Cg	W, Cg
*Perlo 4*	x	x	x	x	x	Al, Cg	W
*Perlo 5*	x	x	x	x	x	Og	W
*Perlo 6*	x	x	x	x	x	Al, Og, Cg	W
Pezzo Grande 2*	x	x	x	x	Vy, Al	x	x
*Pian de Fossa 10*	x	x	x	x	x	Cg	W
*Pian de Fossa 2*	x	x	x	x	x	Cg	W, Cg
*Pian de Fossa 3*	x	x	x	x	Al	Cg	W, Cg
*Pian de Fossa 4*	x	x	x	x	Al, Cg	Al, Vy, Cg	W
*Pian de Fossa 5*	x	x	x	x	Cg	Cg	W
*Pian de Fossa 6*	x	x	x	x	Cg	Cg	W
*Pian de Fossa 7*	x	x	x	x	Cg	Cg	W
*Pian de Fossa 8*	x	x	x	x	Cg	Cg	W
*Pian de Fossa 9*	x	x	x	x	x	Og	W
*Pian di Casa*	x	x	x	x	x	Al	x
*Pian di Manin*	x	x	x	x	x	Al, M	x
*Piana di Gatella*	x	x	x	x	Cg	x	x
*Piano del Ponte rotto*	x	x	x	x	x	Al	x
*Piazzale*	x	x	x	x	Vy, Al, Cg	Vy, Al	x
*Pignon sottano 1*	x	x	x	x	Al	Al	x
*Pignon sottano 2*	x	x	x	x	Vy, Ar, Al	x	x
*Pignon sottano 3*	x	x	x	x	Al, Vy	Al, Vy	x
*Pignon sottano 4*	x	x	x	x	Vy, Ar, Al	x	x
*Pignon sottano 5*	x	x	x	x	Vy, Al	Vy, Al	x
*Pignon sottano 6*	x	x	x	x	Vy, Al	Vy, Al	x
*Pignon sottano 7*	x	x	x	x	Vy, Al	Vy, Al	x
*Pignon sottano 8*	x	x	x	x	Vy, Al	x	x
*Pilora 2*	x	x	x	x	x	Og	W
*Pilora 3*	x	x	x	x	x	Og	W
*Pilora 4*	x	x	x	x	x	Al	W
*Porcile*	x	x	x	x	Vy, M, Ar, Al	x	x
*Porcinasco*	x	x	x	x	Al	x	x
*Possessione 1*	x	x	x	x	Al	Al, Vy, Cg	Al, Vy, Cg
*Possessione 2*	x	x	x	x	x	Al, Vy	Al, Vy
*Possessione 3*	x	x	x	x	x	Al, Vy	Al, Vy
*Pradiera 1*	x	x	x	x	Ar, Vy, Al	x	x
*Pradiera 2*	x	x	x	x	Vy, Al	x	x
*Pradiera 3*	x	x	x	x	Al	Al	x
*Pradiera 4*	x	x	x	x	Al	Al	x
*Pradiera 5*	x	x	x	x	Al	x	x
*Pradiera 6*	x	x	x	x	Al	x	x
*Prati Grandi 1*	x	x	x	x	x	Al	x
*Prati Grandi 2*	x	x	x	x	x	Al	x
*Prati Grandi 3*	x	x	x	x	x	Al	x
*Prato Longo 1*	x	x	x	x	Al, Cg	Al	x
*Prato Longo 2*	x	x	x	x	Al	Al	x
*Prato Longo 3*	x	x	x	x	x	Al	x
*Rivone 1*	x	x	x	x	Cg, Al, Vy	Cg, Al, Vy	x
*Rivone 2*	x	x	x	x	x	Al	x
*Rivone 3*	x	x	x	x	x	Al, Vy	x
*S. Antonio 2*	x	x	x	x	x	Og	W
*S. Antonio 3*	x	x	x	x	x	Og	W
*Saggiano 1*	x	x	x	x	Cg	x	W
*Saggiano 2*	x	x	x	x	Al, Cg	x	W
*Saggiano 3*	x	x	x	x	W	x	W
*Saggiano 4*	x	x	x	x	Cg, W, Al	x	W
*Saramazzo 1*	x	x	x	x	Al, Vy	Al, Vy	Al, Vy
*Saramazzo 2*	x	x	x	x	Al, Og	Al	Al
*Saramazzo 3*	x	x	x	x	Al, Vy	Al, Vy	Al, Vy
*Saramazzo 4*	x	x	x	x	Al, Vy	Al, Vy	Al, Vy
*Saramazzo 5*	x	x	x	x	Al, Vy	Al	Al
*Saramazzo 6*	x	x	x	x	x	Al	Al
*Saramazzo 7*	x	x	x	x	x	Al, Vy, Cg	W
*Saramazzo 8*	x	x	x	x	x	Al, Vy	Al, Vy
*Saramazzo 9*	x	x	x	x	x	Al, Vy	Al, Vy
Scandolara 2*	x	x	x	x	x	Cg, W	W
Scandolara 3*	x	x	x	x	x	W	W
Scandolara 4*	x	x	x	x	x	W, Cg	W
Scandolara 5*	x	x	x	x	x	W	W
*Scoglio del Zer*	x	x	x	x	Ar (walnut)	x	x
*Selva*	x	x	x	x	W	x	x
*Serralunga*	x	x	x	x	x	W	x
*Suasca*	x	x	x	x	x	Cg, W	x
*Taramasco 1*	x	x	x	x	Cg	x	x
*Taramasco 2*	x	x	x	x	Al	x	x
*Termini*	x	x	x	x	Al	x	W
*Tra la Serra*	x	x	x	x	Al, Vy	Al, Vy	x
*Tra le coste 1*	x	x	x	x	Vy, Ar, Al	W	x
*Tra le coste 10*	x	x	x	x	x	Al, Cg	x
*Tra le coste 11*	x	x	x	x	x	Cg, Al	x
*Tra le coste 2*	x	x	x	x	Al	Al	x
*Tra le coste 3*	x	x	x	x	Vy, Ar, Al	Al	x
*Tra le coste 4*	x	x	x	x	Al	Al	x
*Tra le coste 5*	x	x	x	x	Cg, Vy	Cg	x
*Tra le coste 6*	x	x	x	x	Cg, W	Cg	x
*Tra le coste 7*	x	x	x	x	Cg	Cg	x
*Tra le coste 8*	x	x	x	x	Vy, M, Al	Vy, Cg	x
*Tra le coste 9*	x	x	x	x	x	Al	x
*Tra le coste di sopra*	x	x	x	x	x	Cg	x
*Tra le coste di sotto*	x	x	x	x	x	Al, Cg	x
*Tra le vigne*	x	x	x	x	x	Cg, W	x
*Trombassa*	x	x	x	x	x	Cg, W	x
*Va' 2*	x	x	x	x	Cg, Og	Cg, Og	x
*Va' 3*	x	x	x	x	Cg	Cg, W	x
*Va' 4*	x	x	x	x	Og	Og	x
*Va' 5*	x	x	x	x	Al	W	x
*Va' 6*	x	x	x	x	Cg, Og	W	x
*Va' 7*	x	x	x	x	Vy, W	W	x
*Va' 8*	x	x	x	x	Al, Og	Og	x
*Va' 9*	x	x	x	x	Og	Og	x
*Va' 10*	x	x	x	x	Vy, Ar, Al	x	x
*Va' 11*	x	x	x	x	Al, Og	Al, Og	x
*Val del Gallo 1*	x	x	x	x	Vy, Ar, Al	x	x
*Val del Gallo 2*	x	x	x	x	Vy, Ar, Al	x	x
*Val del Gallo 3*	x	x	x	x	Vy, Ar, Al	x	x
*Val del Gallo 4*	x	x	x	x	Al	x	x
*Val del Rio*	x	x	x	x	Al, W	x	x
*Val di Franeta 1*	x	x	x	x	W	W	W
*Val di Franeta 2*	x	x	x	x	x	W	W
*Val di Becco 1*	x	x	x	x	Cg, W	x	x
*Val di Becco 2*	x	x	x	x	W	x	x
*Valdelloro 1*	x	x	x	x	Cg	x	W, Cg
*Valdelloro 2*	x	x	x	x	W	W	W
*Valdelloro 3*	x	x	x	x	W	W	W
*Valdelloro 4*	x	x	x	x	W	x	W
*Valdelloro 5*	x	x	x	x	W, Cg	W, Cg	W
*Valdelloro 6*	x	x	x	x	W, Cg	W, Cg	W
*Valdelloro 7*	x	x	x	x	Cg, W	W	W
*Valetti*	x	x	x	x	x	W	W
*Valle*	x	x	x	x	x	Al	x
*Valle della Vecchia*	x	x	x	x	Cg	Cg, W	W
*Valle di Vallone*	x	x	x	x	x	Cg, W	x
*Valle Grande 1*	x	x	x	x	x	W	x
*Valle Grande 2*	x	x	x	x	x	W	x
*Vanedi 1*	x	x	x	x	Al, Vy	M	x
*Vanedi 2*	x	x	x	x	x	Al	x
*Vanzola*	x	x	x	x	x	W	x
*Varcava 1*	x	x	x	x	Cg	x	W
*Varcava 2*	x	x	x	x	W	x	W

Inventories, ordered for every toponym, of the Municipality of Pignone, for the years 1878, 1933 and recent times.

Analysis of aerial photographs and field surveys allowed the investigation of current land use (crops, natural vegetation, buildings). Ancient toponyms listed in the inventories were compared with current ones that can be found in the cadastral map of the Municipality of Pignone. A complete correlation between ancient and current toponyms does not exist: the former, in part, have been lost; in some cases, more than one type of crop exists for a single toponym.

**Figure 2:**
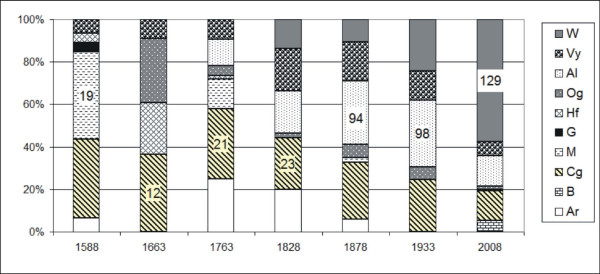
**Frequency crop types**. Percent frequency of crop types calculated for all toponyms.

The antique inventories were written in ancient Italian. Hence the following words, which were found singly or together and linked to toponyms or individually, were annotated (Table 1, Table 2, Figure 2):

- Arboretum (Ar): field planted, probably with fruit trees, such as walnut (*Juglans regia *L.), fig (*Ficus carica *L.), apple (*Malus domestica *(Borkh.) Borkh.), etc. [18,19];

- Buildings (B): area occupied by recent buildings (houses, parking, ways, etc);

- Chestnut grove (Cg): anthropogenic woods of chestnut (*Castanea sativa*), frequently in terrace cultivation.

- Meadow (M): land, probably held for forage production;

- Grassland (G);

- Hempfield (Hf): farmland for hemp (*Cannabis sativa *L.);

- Olive grove (Og): land planted with olives (*Olea europaea *L.), often in terrace cultivation;

- Woodland (W): vegetation to forest (*Quercus *spp., *Pinus pinaster*, *Ostrya carpinifolia*, *Fraxinus ornus*, *Salix *spp., etc.);

- Arable land (Al): very probably crops with wheat (*Triticum *spp.), barley (*Hordeum vulgare *L.) and rye (*Secale cereale *L.); in more recent times, maize (*Zea mays *L.) or vegetables, such as broad bean (*Vicia faba *L.), pea (*Pisum sativum *L.) and chick pea (*Cirer arietinum *L.) [18,19].

- Vineyard (Vy): land planted with vines (*Vitis vinifera *L.).

The percent frequency of all crop portions (type) were computed for each inventory (Figure 2). The relationship between the total number of fields (referred to as toponyms) and the total number of crop portions (for all fields) have been calculated for each inventory as an indicator of crop uniformity (CU).

The botanical nomenclature used in this paper follows [20].

## Results

**Figure 3:**
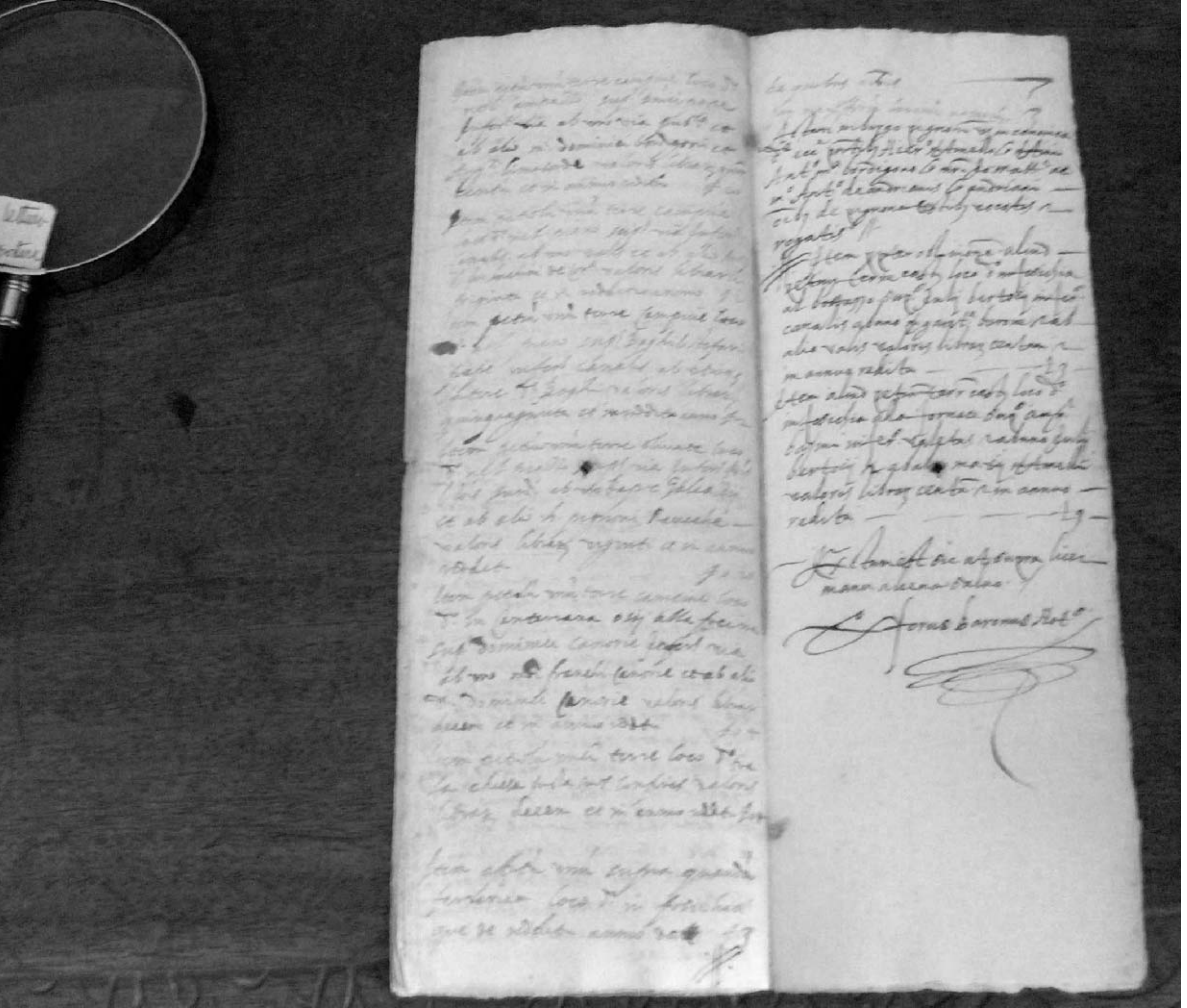
**Ancient document**. An ancient inventory of the Parish of St. Mary in Pignone conserved in the Archivi Vescovili Lunensi of Sarzana.

In the "Niccolo V" Lunense Episcopal Archive of Sarzana, four inventories related to the Parish of St. Mary in Pignone were found and transcribed (Figure 3). The first archival documents date back to 1588; the next date back to 1663, 1763 and 1828 [12-15].

### Year 1588

Fields for forage production (meadow) and chestnut grove prevail (Tab. 1, Figure 3). Other crop types are scarce. The relationship between the number of fields (38) and the number of crop portions (CU: 38/46) is 0.83.

### Year 1663

Chestnut groves prevail, with a small decrease in the total number of fields from 16 (in 1588) to 12. In the XVII century, olive cultivation, not mentioned in the previous inventory, seems to have become extensive. Hemp cultivation increases (from 1 to 8 fields). Three crops cultivated on the vine are present. The CU indicator is 1 (33/33), as all inventoried fields are utilized for only one crop type.

### Year 1763

Chestnut groves prevail in this period as well. A fair number of fields with mixed cultures are present with chestnut groves, arboretums, vineyards and arable lands. There is a decrease in areas planted exclusively with olive crops. The CU indicator is 0.56 (36/64).

### Year 1828

Mixed cultures with vine, arboretum and arable land prevailing. There was a substantial decrease in fields with exclusive chestnut production, but increased mixed areas with chestnut groves and woods. The CU indicator is 0.43 (41/95), the lowest value among the inventories studied.

In the State Archive of La Spezia, two inventories related to the Municipality of Pignone were found and transcribed. The archival documents date back to 1878 and 1933 [16,17].

### Year 1878

Arable land seems to be the main kind of crop, along with chestnut groves with an increase with respect to the previous (ecclesiastic) inventory. Woodlands are almost stable. On the other hand, the relationship between the number of fields and the number of crop (CU) portions increased to 0.61 (194/316).

### Year 1933

Arable land prevail. There was a substantial decrease in fields with chestnut groove and a increase of woods. The CU indicator is 0.73 (226/311).

### Current

To monitor the present landscape and crops, field surveys were carried out with the help of aerial photographs and the cadastral map of the Pignone territory. This allowed us to compare present and past situations linked to specific toponyms. The majority of ancient fields with chestnut groves and olive groves have been abandoned and partially reforested with cluster-pine woodland or mixed coppiced woodland (oaks, chestnuts, European hop-hornbeam, etc.). Some areas now have buildings.

At the other sites in Pignone, not linked to the toponyms noted in the inventories, a general reforestation has been surveyed. Vineyards and olive groves at present have been on the whole abandoned. The few arable lands consist of maize or vegetables. The CU indicator is 0.75 (168/224).

## Discussion

Ecclesiastic institutions were among the main landowners in Europe until the end of the XIX century. For this reason, such inventory data gives us qualitative and semiquantitative information regarding the types of crop change throughout time in an area of eastern Liguria. After the XIX century, data from the inventories of municipalities and from forest inventories are available for the same territory. Such information is in agreement with historical knowledge related to the study area and to other Mediterranean hill/mountain areas.

The chestnut, in historical times, was spread by Romans as plant for food. However, it is well documented that starting in the Middle Ages chestnut cultivation spread throughout the Mediterranean hill/mountain areas of Italy (then in Val di Vara), replacing part of the holm-oak woods [21-23]. In Val di Vara, domestication of the woodland chestnut tree spread during the XIV century, along with a strong increase in population [24]. Our data confirm such assertions and show that the main subsistence means in the study area from the XVI century to the first half of the XIX century was chestnut, and secondarily meadow in 1588, hempfield and olive in 1663, arboretum in 1763 and vine in 1828. In the study area in 1878 and 1933, arable land prevailed along with chestnut groves. High values of the CU indicator have evidenced few diversified crops in the XVI and XVII centuries. Crop diversification increased until the first half of the XIX century (low values of the CU indicator), then it decreased again (high values of the CU indicator).

In addition, in other Mediterranean regions, chestnut has been a tree historically cultivated and utilized as food, but also for a variety of other practices [23,25]. For example, chestnut could be utilized around crops in lines together with other trees as a barrier to wind, to stabilize soil and to support the harvest of leaves and branches for livestock [26]. In vineyards, vines were generally sustained by chestnut poles arranged in rows or pergolas [26].

In the study area and during some periods, a lesser part was played by olives and vines in agricultural activities. On the contrary, in the warmer areas of the Mediterranean basin close to the coast, olive groves and vineyards were the main crop types [5].

General investigation of the Ligurian Republic dated back to 1799 [27] and supplied data regarding forest vegetation and crop types in Val di Vara (some locations). For example, in the nearby Val di Pino, holm-oak and chestnut crops were present. In other locations in Val di Vara (San Pietro Vara and Varese Ligure) fields of chestnut, turkey-oak (*Quercus cerris *L.), downy-oak, European hop-hornbeam, beech (*Fagus sylvatica *L.), willow trees (*Salix *spp.) and olive trees were noted [27,28]. Crops with major production indices included chestnut, followed by wine, olive oil, wheat, rye, fruits, figs, legumes, potatoes (*Solanum tuberosum *L.), and vegetables [18,19]. Particularly interesting is the earlier presence of cultivated potatoes in Val di Vara. In Italy, the potato was introduced in Genova around 1585 by Carmelite Friars from Spain and Portugal [29]. However, the potato had begun to spread throughout all of Europe, and in eastern Liguria starting from the second half of the XVIII century [30,31]. Around Genova to the Val di Vara territory, the variety "Quarantina bianca" was cultivated [29]. The progressive increase in the cultivation and utilisation of potato and maize is considered one of the causes of the decrease in nutritional importance of the chestnut tree starting from the second half of the XIX century [32]. Our data support this assertion, as in the inventories of 1878 and 1933 arable land (Al) were the main crop type.

Starting from the XIX century (inventory of 1828), there was an increase of woodlands. This was very probably due to a transformation in the socio-economic conditions and also to the production of coal inside charcoal pits [22,33]. This practice disappeared everywhere after the Second World War, but gaps produced by the old charcoal spaces persist and are still visible in the woods [26]. In eastern Liguria (also in the Pignone territory), there has been increased extractive activities in this century in several modest mines for industrial, energy or building purposes, such as pyrite, kaolin, lignite, and sandstone. Such activity locally favoured the exploitation of the woods in the areas close to the mines [34].

Since the first half of the XIX century, chestnut ink disease, caused by *Phytophthora cambivora *and *P. cinnamomi *has become widespread in Europe [35]. In Italy and particularly in eastern Liguria, ink disease and chestnut disease (cancer of the bark) gravely damaged chestnut groves starting from the end of the XIX century until about 1950 [36-38].

Before the XX century, the agricultural system in the study area mainly produced food for self-sustaining local communities, parishes and landowners, and only occasionally for external commerce [26].

During the XX century, chestnut groves in the study area significantly decreased in importance. Such trends have been evidenced in upper Val di Vara [24]: in 1938, the chestnut woods (coppiced and trees) covered about 60% of the territory. After the Second World War, in Val di Vara there were strongly augmented plantations of the most degraded woodland (erosion) with coniferous trees [39]. In 1993, most of the territory was forested by mixed woodlands, turkey-oak woodlands and plantations (*Pinus *spp.), and only 15% of the area was occupied by chestnut coppice [24].

The recent forest census of the Liguria Region revealed that only about 22% of the forest surface of the whole Province of La Spezia is occupied by chestnut (comprising coppice); mixed coppiced woodland and cluster pine woodland represent 21.8% and 15.2% respectively [40]. The same forest census showed that in the Pignone territory, chestnut coppice represents 20%, mixed woodland 27.60% (oaks, chestnuts, European hop-hornbeam, etc.), cluster pine woodland 13.1%, olive grove 2.5%, and other cultivated lands 9.7% [40].

In this context, the traditional elements of the rural landscape, in terms of chestnut groves and olive groves (terraces, stonewalls, etc.), have been not preserved, but rather have deteriorated or been destroyed [5]. We can assert that agriculture abandonment and changing socio-economic conditions over the last 50 years have favoured a strong increase in cluster-pine woodland or mixed coppiced woodland (oaks, chestnuts, European hop-hornbeam, etc.) and partially in evergreen sclerophyllous forest and shrubland at the expense of cultivated areas, especially those with chestnuts and olives [7]. A similar trend has been observed for coastal areas of eastern Liguria, where vineyard, the dominant crop type, has been mainly substituted by cluster pine woodland [40,41]

As evidenced in other Mediterranean hill/mountain areas, this has lead to a simplification of the landscape (an increase in the CU indicator) due to abandonment of agricultural practices and reforestation [42,31].

## Conclusion

This study has examined crop/land cover changes during the last five centuries in an area of eastern Liguria (Italy) through examination of archival documents taken from ecclesiastic and municipal inventories, and current field surveys. In accordance with other studies conducted in the Mediterranean region, we show evidence of the traditional use of chestnut as one of the main source of food in the hill/mountain areas of eastern Liguria in the XVI, XVII and XVIII centuries. This work has highlighted how ancient documents, which are at present not generally used in ethnobotanical studies, can be very useful for enhancing the knowledge of agricultural and livestock practices and of subsistence methods favoured by local populations during a particular time. This study has also shown that inventories (but also other types of documents) are very useful for the historical reconstruction of the landscape by means of relative comparison of cultivated fields, anthropogenic woods and naturally reforested areas.

## Competing interests

The authors declare that they have no competing interests.

## Authors' contributions

RG and EG found and transcribed the ancient document. RG and SG wrote the paper and prepared the tables and figures. All authors read and approved the final manuscript.
